# Preclinical and Clinical Epigenetic-Based Reconsideration of Beckwith-Wiedemann Syndrome

**DOI:** 10.3389/fgene.2020.563718

**Published:** 2020-09-15

**Authors:** Chiara Papulino, Ugo Chianese, Maria Maddalena Nicoletti, Rosaria Benedetti, Lucia Altucci

**Affiliations:** Department of Precision Medicine, Università degli Studi della Campania “Luigi Vanvitelli”, Naples, Italy

**Keywords:** Beckwith-Wiedemann syndrome, rare diseases, cancer predisposition, epigenetics, metabolic disorders, DNA methylation, monozygotic twins

## Abstract

Epigenetics has achieved a profound impact in the biomedical field, providing new experimental opportunities and innovative therapeutic strategies to face a plethora of diseases. In the rare diseases *scenario*, Beckwith-Wiedemann syndrome (BWS) is a pediatric pathological condition characterized by a complex molecular basis, showing alterations in the expression of different growth-regulating genes. The molecular origin of BWS is associated with impairments in the genomic imprinting of two domains at the 11p15.5 chromosomal region. The first domain contains three different regions: insulin growth like factor gene (*IGF2*), *H19*, and abnormally methylated DMR1 region. The second domain consists of cell proliferation and regulating-genes such as *CDKN1C* gene encoding for cyclin kinase inhibitor its role is to block cell proliferation. Although most cases are sporadic, about 5–10% of BWS patients have inheritance characteristics. In the 11p15.5 region, some of the patients have maternal chromosomal rearrangements while others have Uniparental Paternal Disomy UPD(11)pat. Defects in DNA methylation cause alteration of genes and the genomic structure equilibrium leading uncontrolled cell proliferation, which is a typical tumorigenesis event. Indeed, in BWS patients an increased childhood tumor predisposition is observed. Here, we summarize the latest knowledge on BWS and focus on the impact of epigenetic alterations to an increased cancer risk development and to metabolic disorders. Moreover, we highlight the correlation between assisted reproductive technologies and this rare disease. We also discuss intriguing aspects of BWS in twinning. Epigenetic therapies in clinical trials have already demonstrated effectiveness in oncological and non-oncological diseases. In this review, we propose a potential “epigenetic-based” approaches may unveil new therapeutic options for BWS patients. Although the complexity of the syndrome is high, patients can be able to lead a normal life but tumor predispositions might impair life expectancy. In this sense epigenetic therapies should have a supporting role in order to guarantee a good prognosis.

## Introduction

Epigenetic alterations play a crucial role in both cancer and non-oncological diseases (Mau and Yung, 2014) regulating DNA methylation, histone modifications (Nowacka-Zawisza and Wisnik, 2017) and micro-RNA expression that ultimately determines gene expression (Abi Khalil, 2014).

Some rare congenital diseases present alterations in epigenetically regulated genes (Niculescu and Lupu, 2011), providing the rationale to interfere via an epigenetic rebalance to mitigate/overcome these conditions (Nguyen, 2019). BWS is an example of a complex disease characterized by genetic and epigenetic aberrations on chromosomal region 11p15.5 comprising a telomeric and a centromeric domain, both regulated by genomic imprinting (Gomes et al., 2009). BWS is characterized by juvenile abnormal overgrowth (Choufani et al., 2010; Eggermann et al., 2015), affecting 1 child in 10,340–13,700 live births worldwide (Mussa et al., 2013). The associated phenotype may have a range of manifestations (Maas et al., 2016; Dawkins et al., 2018), although the most common clinical features are macroglossia, present in half of the patients with a molecular defect at 11p15.5 (Gaston et al., 2001; Brioude et al., 2018), visceromegalia and abdominal wall defects. Minor features include ear pits, hypoglycemia, nephromegaly, and isolated lateralized overgrowth (Mussa et al., 2016b). BWS is equally incident in males and females, but in monozygotic twins, there is observed an excess in females (Weksberg et al., 2010), and the discordant disease presentation suggests an important epigenetic role (Bliek et al., 2009b).

BWS is considered an imprinting disorder (IDs) affecting growth, development, and metabolism (Netchine et al., 2013) often caused by alterations in imprinting control regions (ICRs) in the parental 11p15.5 region (Abramowitz and Bartolomei, 2012; Begemann et al., 2012; Abi Khalil, 2014). ICRs impairment leads to abnormal methylation state deregulating genes as *CDNK1C, H19, IGF2*, and *KCNQ1OT1* involved in growth, so provoking the onset of BWS features (Wang et al., 2020). The expression/inactivation of a gene is related to differentially methylated regions (DMRs).

The majority of IDs such as BWS present the same four classes of molecular alterations lead to imbalanced gene expression: uniparental disomy (UPD), epimutation (aberrant methylation marks), copy number variations (CNVs) and point mutations in imprinted genes. Since DNA methylation marks are transmitted through generations, an interesting approach for BWS resides in the epigenetic-regulated inheritance study, which is still in an early phase. In that case, the major limitation for it to be carried on is due to the low patients’ number (Nguyen, 2019). For this reason, multi-institutional collaborations are required in order to reach a statistically significant number of patients for the constitution of an observational cohort or a clinical trial (Griggs et al., 2009). Moreover, the low BWS prevalence requires special combined efforts to improve diagnosis, care, and prevention (Ayme and Schmidtke, 2007). In order to overcome this limitation and trying to boost patient care, the Coordination of Rare Diseases at Sanford (CoRDS) started in 2013 a program (NCT01793168) providing a centralized- international patients registry for all rare diseases. This program helps in the identification, advance treatments, and therapies on a large cohort of patients.

## Role of Epigenetic Alterations in BWS

**FIGURE 1:**
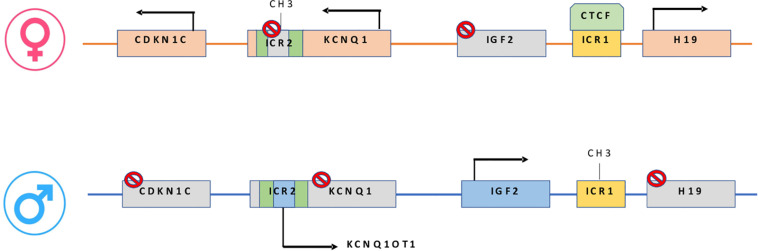
On the maternal allele: CTCF binding H19/IGF2:IG-DMR, prevents IGF2 from accessing the shared enhancers located on the H19 side. On the paternal allele: H19/IGF2:IG-DMR methylation prevents CTCF from binding, allowing IGF2 to engage the enhancers and silencing the H19 promoter. In maternal KCNQ1OT1:TSS-DMR, methylation induces KCNQ1-CDKN1C expression while paternally unmethylated state represses KCNQ1-CDKN1C expression and increase ncKCNQ1OT1 transcription.

**FIGURE 2:**
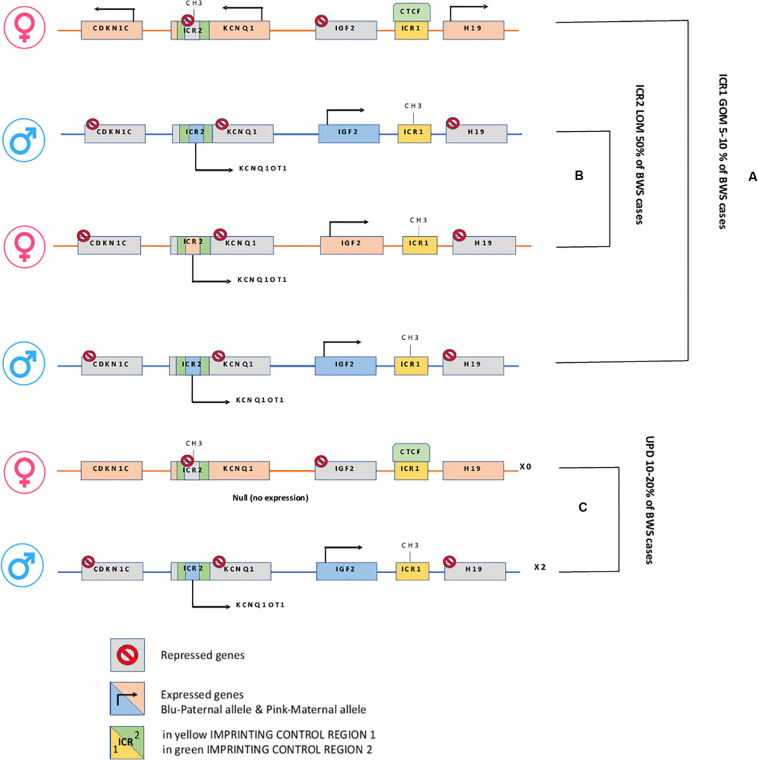
H19/IGF2:IG-DMR and KCNQ1OT1:TSS-DMR. **(A)** In Maternal allele H19/IGF2:IG-DMR GOM leads to overexpression of the growth factor IGF2. **(B)** In Maternal KCNQ1OT1:TSS-DMR LOM, the ncRNA *KCNQ1OT1* is expressed while *cis*-linked genes are repressed. **(C)** Paternal uniparental disomy.

Epigenetic alterations associated with BWS are present on two different domains, independently methylated in the 11p15.5 gene cluster (Krzyzewska et al., 2019): *H19/IGF2*:IG-DMR (intergenic differentially methylated region) and *KCNQ1OT1*:TSS-DMR (transcription start site differentially methylated region) (Figure 1). The first region is also known as ICR1 consisting of two main imprinted genes, *IGF2* and *H19. IGF2* is paternally expressed and it encodes s for a fetal growth factor implicated in BWS pathogenesis. *H19* is a maternally expressed allele, non-coding RNA, and its function is still unclear, although it has been postulated a tumor suppressor role (Cai and Cullen, 2007; Raveh et al., 2015). *IGF2* deregulation determines overgrowth and region-specific tumor development in BWS (Schofield et al., 2001). *IGF2* and *H19* genes are oppositely imprinted with an enhanced competition (Maher and Reik, 2000). The *H19/IGF2*:IG-DMR domain works as a chromatin insulator regulating the expression of *IGF2* and *H19* genes. The chromatin insulator is located 2 Kb upstream of the *H19* gene and displays CTCF binding sites (Hark et al., 2000). Once CTCF binding is prevented, the enhancers can interact with *IGF2* promoters. Oppositely the unmethylated maternal allele allows CTCF binding to the insulator elements blocking the downstream enhancers of *H19* to access the *IGF2* promoter (Soejima and Higashimoto, 2013) (Figure 2A). H19/IGF2:IG-DMR-specific histone marks have also been reported: the methylated region presents H3K9me3 and H4K20me3, whereas the unmethylated area carries H3K4me2/3 and H3/H4 acetylated (Nativio et al., 2011). In 5-10% of BWS patients observed with a gain of methylation (GOM) of the H19/IGF2: IG-DMR on the unmethylated maternal allele (Figure 2B) (Eggermann et al., 2014; Brioude et al., 2018). The aberrant methylation is also characterized by variations in histone signatures. Accessible modification of H3K9ac and bivalent H3K4me2/H3K27me3 are converted to the repressive H3K9me3 and H4K20me3 (Nativio et al., 2011). These chromatin alterations prevent CTCF binding to the maternal *H19/IGF2*:IG-DMR, thus enabling the enhancers to access the *IGF2* promoter and leading both to a biallelic expression of *IGF2* and reduced expression of *H19*. In detail, *H19/IGF2*:IG-DMR is characterized by 59-bp elements hD1 and hD2, composed mainly of two Sox-Oct motif-like sequences (SO motifs) and a single Oct motif (rO) and are accompanied by triple-repeat sequences containing CBS1-3 or CBS4-6 (also known as CBSs). Recently, by using transplantation in mice, it was reported that H19/IGF2:IG-DMR methylation status is regulated by the CBSs and SO motifs (Hori et al., 2012). Interestingly in BWS patients, SO motifs are mutated or deleted. Three Oct motifs variants found in BWS patients disrupt hD1-dependent DNA demethylation and cause the stack of methylation (Kubo et al., 2020). Single nucleotide substitutions in hD1 Oct motifs present in BWS patients show hypermethylation in *H19/IGF2*:IG-DMR coupled with histone modifications: maternal aberrant DNA methylation is connected with reduction of H3K4me2 and H3K9ac and increase of H3K9me3 and H3K27me3 (Kubo et al., 2020). *KCNQ1OT1*:TSS-DMR contains six different imprinted genes: *KCNQ1*, *KCNQ1OT1*, *CDKN1C*, *SLC22A18*, *TSSC3*, and *PHLDA2*. *CDKN1C* is a maternally expressed gene encoding for a cell cycle regulator, a cyclin-dependent kinase inhibitor. It regulates in a negative manner cell proliferation. *KCNQ1OT1* is a paternally expressed long non-coding RNA capable to inhibit the expression of genes in the domain *in cis*. The KCNQ1OT1:TSS-DMR of this domain is localized in the intron 10 of the *KCNQ1* gene, and it is methylated on the maternal but not on the paternal allele; thus the unmethylated paternal one allows the transcription of *KCNQ1OT1*:TSS-DMR, preventing the *CDKN1C* and *KCNQ1* gene expression. In mice, this DMR interacts with G9a and the PRC2 complex leading to repressive histone modifications such as H3K9me3 and H3K27me3. The methylated *KCNQ1OT1*:TSS-DMR form present on the maternal allele prevents the transcription of the long non-coding RNA, *KCNQ1OT1*, allowing the expression of several genes present in the domain such as *CDKN1C*. Loss of methylation (LOM) on the maternal allele occurs in 50% of BWS patients (Eggermann et al., 2014; Brioude et al., 2018). This molecular defect is accompanied by changes in histone modifications, such as the loss of H3K9me2 (Soejima and Higashimoto, 2013). LOM is responsible for the biallelic expression of the *KCNQ1OT1* transcript, which reduces the *CDNK1C* expression provoking BWS phenotype. *CDNK1C* gene acquires biallelic methylation in some tumors and in 5% of BWS cases, this gene presents point mutations (Du et al., 2003). Another molecular alteration is UPD(11)p present in 10-20% of BWS patients (Eggermann et al., 2014; Brioude et al., 2018) (Figure 2C). UPD condition results when both chromosomes -or part of them- is/are inherited from one parent. In BWS, the UPD is paternal and involves the 11p15 region implying no maternal contribution for it. UPD is related to mitotic recombination during embryonic development. BWS patients with UPD(11)pat show mosaicism suggesting to be a post-zygotic event. The mosaicism level of UPD in different tissues is strongly associated with the pathological phenotype. The size of the UPD region or the level of mosaicism are not correlated with the severity of the disorder (Cooper et al., 2007). Patients with a mosaic UPD(11)pat for the entire chromosome 11 present clinical features comparable to UPD(11)pat cases restricted to a small part of 11p. BWS patients present UPD(11)pat isodisomic, thus determining increased *IGF2* expression and reduced *H19* expression (Wang et al., 2020). In some BWS patients, it is reported that β*2SP* gene, a TGF-β/Smad3/4 adaptor protein, a potent tumor suppressor is epigenetically silenced, thus observing a loss or markedly decreased expression of β*2SP* related to aberrant DNA methylation of CpG islands around its promoter region (Yao et al., 2010).

## BWS as Multi-Locus Imprinting Disorder

Some BWS patients but also other imprinting disorder cases present additional methylation defects at different imprinted loci determining a pathological condition known as multi-locus imprinting disturbance (MLID) that potentially alters the expression of multiple imprinted gene clusters (Bens et al., 2016; Sanchez-Delgado et al., 2016). The MLID frequency is related to the type of imprinting disorder and appears to vary depending on the technology sensitivity used to analyze and on Imprinted Differentially Methylated Regions (IDMRs) investigated (Poole et al., 2013). Currently, BWS shows at frequencies around 50% in cases with LOM at KCNQ1OT1:TSS-DMR (Court et al., 2013), while MLID condition is less observed among patients with GOM at H19/IGF2:IG-DMR (Maeda et al., 2014). Some of MLID BWS patients present LOM or GOM at both maternal and paternal IDMRs (Court et al., 2013; Poole et al., 2013) while others have a hypomethylation syndrome restricted to maternally imprinted genes (Boonen et al., 2008; Baple et al., 2011). MLID condition usually presents BWS clinical features but in some cases in patients shows complex or atypical phenotypes, conceivably reflecting the loci and tissues mosaicism (Docherty et al., 2014; Begemann et al., 2018) and probably as a consequence of the dominance of one locus on other (Zhang et al., 1997; Azzi et al., 2009). In a cohort study, MLID was identified only in BWS patients with KCNQ1OT1:TSS-DMR LOM and hypomethylation was found only at maternally iDMRs, a condition described as multiple maternal hypomethylation syndrome (MMHS) (Bliek et al., 2009b; Sano et al., 2016; Fontana et al., 2018). A small number of manuscripts on MLID reported as GOM at paternally methylated iDMRs in BWS patients (Court et al., 2013; Maeda et al., 2014). The most frequently altered iDMRs found in BWS patients with MLID are *PLAGL1*, *GRB10*, *MEST*, *GNAS*, *IGF2R*, and *ZNF331*. From a pathological point of view, usually, MLID BWS patients have a decreased level of body weight compared to the one who characterized by a single molecular defect in the 11p15.5 region. Specifically, MLID patients show features not typical of BWS, such as speech retardation, apnea, and feeding difficulties (Bliek et al., 2009b). MLID-associated clinical signs may also only manifest as patients grow up; therefore MLID analysis after molecular confirmation of a specific ID, could guide a patient-tailored follow-up to track subclinical signs before their manifestation (Bakker et al., 2016). In 2005 for the first time there were reported two patients with MLID presented transient neonatal diabetes mellitus (TNDM) and hypomethylation of both iDMRs of *KCNQ1OT1* gene (Arima et al., 2005) and of *PLAGL1*, an antiapoptotic (Shuman et al., 2006) gene located at chromosome 6q24 (Bliek et al., 2009b). A further broad analysis revealed that the MLID prevalence is higher in BWS cases than in other imprinting disorders (Brioude et al., 2018). However, despite the growing number of studies, the etiology of MLID is still unclear as well as mechanisms underlying the co-regulation of imprinting marks across the genome. However, MLID causative mutations have been identified in members of the NLRP and zinc-finger protein families in few BWS patients (Docherty et al., 2014). Their role in the imprinting process and the pattern of inheritance has yet to be fully elucidated. Molecular characterization of MLID is fundamental not only to define the clinical diagnosis of IDs better but also to evidence common functional networks at the basis of the imprinting genome-wide deregulation.

## Genes Candidates in Overgrowth Feature in BWS

BWS is a childhood cancer predisposition disorder with increased risk of embryonic tumors, predominately Wilms tumor, and hepatoblastoma (Duffy et al., 2018). The chromosome 11p15.5 region contains imprinted genes that are fetal growth regulators (Brioude et al., 2018). The molecular analysis of domain 1 and 2 has identified two candidates related to tumor development in BWS: *CDKN1C* (*p57Kip2*) and *IGF2*. Their aberrations are associated with growth and development disturbances. *IGF2* is a cell cycle regulator gene encoding for a growth factor with different functions in promoting cell growth and proliferation during fetal development (Park et al., 2017). *IGF2* binds three receptors, IGF1 receptor (IGF1R), insulin receptor isoform A (IR-A), and the IGF1R–IR-A hybrid receptor, resulting in a cascade of intracellular events and promoting cell survival and mitogenesis (Alvino et al., 2011). *IGF2* loss of imprinting from DMR dysregulation on the maternal allele increases its signals, thus promoting growth and anti-apoptosis processes (Gallagher and LeRoith, 2010). In addition, the dysregulation of *IGF2* expression has been recently observed also in several tumor onsets such as for breast, ovarian, esophageal, and colorectal cancer, and its presence has been reported and associated with poor prognosis (Livingstone, 2013). *IGF2* is involved in prenatal skeletal muscle growth and in muscle regeneration in adults. It has been studied using *in vitro* differentiation models and *in vivo* loss of function in mouse models (Park et al., 2017). In mice, the *IGF2* overexpression determines BWS clinical features leading to overgrowth, polyhydramnios, fetal and neonatal lethality, disproportionate organ overgrowth, and macroglossia (Murrell et al., 2004). The *CDKN1C* encodes for a negative regulator of the cell cycle, and its function is to inhibit several Cyclin/CdK complexes. This gene maps in the centromeric region of 11p15, and it is paternally imprinted, thus implying its expression on the maternal allele. *CDKN1C* is considered a tumor suppressor gene. It blocks cell proliferation by inhibiting cell cycle progression, tissue invasion, metastasis, angiogenesis, and promotes apoptosis and cell differentiation (Kavanagh and Joseph, 2011). CDKN1C protein is composed of 316 amino acids assembled in three different domains: the N-terminal domain is fundamental for CdK inhibition, a central repeating sequence, and a C-terminal domain working as the PCNA binding domain homolog to p27Kip1 it interacts with PCNA protein (Stampone et al., 2018).

In BWS patients, the missense variants of *CDNK1C* involve highly conserved amino acids of the N-terminal domain with the CdK inhibitory role, impairing its function or its cellular localization. Until 2014 there were described 33 different point mutations in coding and non-coding regions of the gene (Eggermann et al., 2014). Most of them were missense mutations in frame-shift, leading to the production of a truncated form of the protein (Brioude et al., 2015). 5–10% of BWS patients show pathogenic variants of *CDKN1C* gene on the maternal allele, thus determining its loss of function (Eggermann et al., 2014; Brioude et al., 2018). Several *CDKN1C* gene point mutations have been identified, 40% with a BWS family history (Romanelli et al., 2010; Eggermann et al., 2014; Brioude et al., 2018). In the Human Gene Mutation Database, 65 different variants are reported in BWS patients exhibiting different clinical features including polydactyly, extra nipple, genital anomalies, and cleft palate, strongly dependent on epigenetic/chromosomal abnormalities at chromosome 11p15.5. BWS patients carrying *CDKN1C* mutations show a higher frequency of abdominal wall defects and omphalocele (Brioude et al., 2015), whereas mice lacking of a maternal copy of the *CDKN1C* gene present a phenotype close to BWS with gastrointestinal tract abnormalities or exomphalos (Yan et al., 1997; Zhang et al., 1997). *CDKN1C* mutations have also been correlated to several types of cancer, such as colorectal, lymph-hematologic, breast cancer (Larson et al., 2005), and it emphasizes the presence of commonalities features between BWS and cancer predisposition.

## The Clinical Spectrum of BWS

**FIGURE 3:**
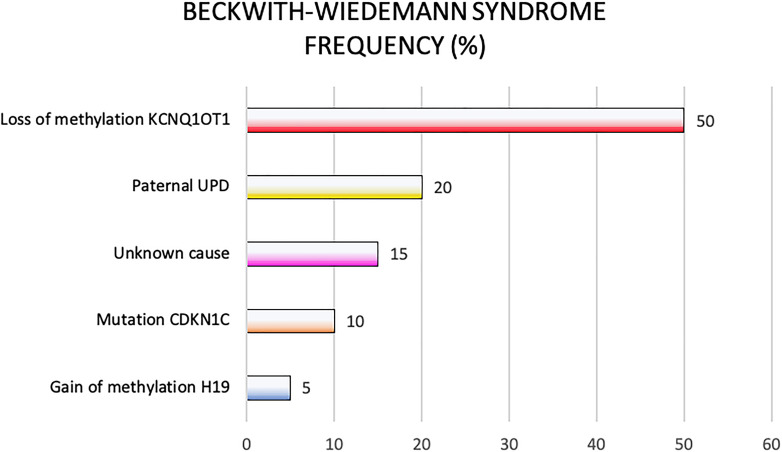
Beckwith-Wiedemann syndrome frequency of molecular defects.

Genetic and epigenetic changes frequently lead to different clinical phenotypes reported in the clinical criteria applied for the BWS definition. In Figure 3 are reported genetic and epigenetic abnormalities in association with BWS patients’ recurrence.

An abnormal epigenetic variability may cause the onset of pathological mosaic states, one with a normal genotype (Zhang et al., 1997) and the other one carrying modified epigenetic information (Sazhenova and Lebedev, 2008). Thus in 2018, due to this high complexity, the Consensus Group introduced the concept of BWS spectrum (BWSp) including patients with a clinical diagnosis of BWS with or without an epigenetic alteration at the 11p15 locus, patients with “atypical BWS” (a condition defined by fewer cardinal and suggestive features than those needed for a BWS clinical diagnosis) and an epigenetic change at the BWS locus and patients with “isolated lateralized overgrowth.” The accurate identification of clinical aspects is crucial for the diagnosis: cardinal features include macroglossia, exomphalos, lateralized overgrowth, multifocal Wilms tumor, prolonged hyperinsulinism, and distinct pathological findings are unique to BWS. Suggestive features are less specific but may help the clinical diagnosis and the indication for molecular testing. These features are birth weight (>2SDS above the mean), facial nevus simplex, polyhydramnios and/or placentomegaly, ear creases and/or pits, transient hypoglycemia (lasting < 1 week), typical BWSp tumors (neuroblastoma, rhabdomyosarcoma, unilateral Wilms tumor, hepatoblastoma, adrenocortical carcinoma or pheochromocytoma), nephromegaly and/or hepatomegaly, umbilical hernia and/or diastasis recti (Brioude et al., 2018). The consensus criteria for clinical diagnosis apply a scoring system: each cardinal feature gets two points while each suggestive feature gets one point. For the clinical diagnosis, a score ≥ 4 is required, and the molecular confirmation of 11p15.5 anomalies needs to be applied. A genetic test is also required with a score ≥ 2 with further evaluation of a BWS expert (Brioude et al., 2018).

## BWS Dedicated Molecular Investigations and Prenatal Test

BWS has genetic and epigenetic abnormalities, and it is phenotypically associated with different clinical aspects; therefore, the analysis of the molecular features of patients is essential for their management and treatment. First molecular testing procedure is usually applied evaluating mosaicism using DNA from blood leukocytes; then, the analysis may be implemented on additional samples such as buccal swabs, skin fibroblasts, or cells of mesenchymal origins. The procedure evaluates H19/IGF2:IG-DMR and KCNQ1OT1:TSS-DMR methylation levels as well as DMR copy number variations (CNVs) (Brioude et al., 2018). The most common diagnostic test for this purpose is Methylation-Specific Multiplex Ligation-dependent Probe Amplification (MS-MLPA) able to detect the percent of methylation and DMR copy number status simultaneously (Priolo et al., 2008; Scott et al., 2008; Wang et al., 2020). Low-level mosaicism patients require more sensitive methylation-specific techniques such as MS-PCR, MS quantitative PCR and in particular chromosomal microarray analysis (CMA) can determine the length of the UPD(11)pat region (Coffee et al., 2006; Azzi et al., 2011; Russo et al., 2016). Single gene sequencing of *CDKN1C* mutations can clarify the recurrence risk in family members (Brioude et al., 2018). Chromosome microarray might be required to define the size and the nature of duplication or deletion in the case of CNV (Baskin et al., 2014; Liu et al., 2015), whereas fluorescence *in situ* hybridization (FISH) or karyotyping may also provide the identification of chromosomal translocations (Bi et al., 2013). Prenatal molecular tests may allow handling the disease before the birth despite results reliability, and the ethical issues need to be taken into account (Brioude et al., 2018). This type of analysis requires samples such as chorionic villus (CVS) cells, amniotic fluid (AF) cells or fetal blood cells (native and cultured) (Brioude et al., 2018) but it is essential to consider that CVS cells could have different methylation patterns of the 11p15.5 region from embryonic tissues (Paganini et al., 2015) showing false-positive results due to tissue mosaicism (Brioude et al., 2018). For this reason, it is highly recommended that a multicenter audit of cases in order to implement different methods and get a correct diagnosis (Brioude et al., 2018). Different methods and diagnostic rates might be applied (Eggermann et al., 2016; Brioude et al., 2018). Quantitative PCR from amniocytes and cord blood leucocytes in 15 weeks pregnant women (see also NCT01842659) might result useful to evaluate the imprinting region 11p15.5, the Methylation Index (MI), the Interclass Correlation Coefficient (ICC) and the agreement between these two samples. Biochemical screening results such as elevated levels of free β-human chorionic gonadotropin (hCG) in the first trimester and/or increased α-fetoprotein (αFP) levels in the second trimester (associated with exomphalos) can be associated with BWS in the fetus (Gocmen et al., 2005; Kagan et al., 2015). BWS diagnosis can also result from an ultrasonographic detection of an anterior abdominal wall defect, macroglossia, or, less accurately, from macrosomia, visceromegaly, polyhydramnios, placentomegaly, or pancreatic overgrowth. BWS diagnosis can also be proposed by prenatal ultrasound scan (USS) identifying placental mesenchymal dysplasia, urinary tract abnormalities, cardiac defects, adrenal cysts and masses (Kagan et al., 2015; Brioude et al., 2018). Williams et al., 2005 reported diagnostic scheme based on the identification by ultrasound examination of different findings: at least two main (i.e., an abdominal wall defect, macroglossia, or macrosomia), or one main and two minor findings are required to lead BWS diagnosis (i.e., nephromegaly/dysgenesis, adrenal cytomegaly, aneuploidy/abnormal loci, or polyhydramnios) (Williams et al., 2005).

## Predisposition in Tumor Development: Estimated Cancer Risk in BWS

Epigenetic aberrations effects in BWS phenotype may determine different tumor predisposition (Segers et al., 2012). Numerous pieces of evidence suggest that cancer is strongly related to the patient’s age (Kalish et al., 2016; MacFarland et al., 2018). There is a high risk during the first 4 years of life (11%) reduced to 3% in the 4–10 years-old patients (DeBaun and Tucker, 1998). Screening and diagnostic procedures should be applied for early diagnosis. The screening phase consists of repeated abdominal ultrasound (every 3–4 months during the first 8-10 years of life) and serum α-fetoprotein (aFP) measurement (every 3 months during the first 30 months of life) (Mussa et al., 2019). These procedures are applied to all patients regardless of the molecular diagnosis and the genotype (Zhang et al., 1997).

BWS molecular subtypes are related to tumor predisposition, and each subtype is associated with the development of one (or more) type of cancer (Mussa et al., 2016a). In Table 1 are reported the molecular subgroups and cancer types occurrence.

**TABLE 1:** Association of BWS molecular defects in subgroups and tumor risk frequency.

Molecular defect	Alteration	Frequency of molecular defects	Tumor risk compared with other molecular subgroups
H19/IGF2:IG-DMR HYPERmethylation	Hypermethylation	5–10%	High risk of Wilms tumor
UPD(11)pat	Paternal UPD	20%	High risk of Wilms tumor and hepatoblastoma
KCNQ1OT1:TSS-DMR HYPOmethylation	Hypomethylation	50%	Tumor incidence is lower than the other molecular subgroups and is very variable
CDKN1C mutations	Loss of function mutations	5–10% (Sporadic cases 5%; Familial cases 40%)	Low risk of Wilms tumor

*Adapted from Cooper et al. (2005), Eggermann et al. (2014), Maas et al. (2016), Mussa et al. (2016a), and Brioude et al. (2018).*

The four main molecular subtypes of BWS (KCNQ1OT1:TSS-DMR-LOM, H19/IGF2:IG-DMR -GOM, UPD, and *CDKN1C* mutations) are characterized by specific genotype-phenotype correlation to tumor development risk (Ibrahim et al., 2014; Mussa et al., 2016a). Patients with 11p15 telomeric domain defects (H19/IGF2:IG-DMR -GOM and UPD) have a higher risk of developing cancer compared to cases presenting centromeric aberrations (KCNQ1OT1:TSS-DMR-LOM mutation and *CDKN1C*) (Soejima and Higashimoto, 2013; Maas et al., 2016). The molecular subtype characterization of each BWS case increases those patients at the highest cancer risk focusing on a specific cancer. Subtypes analysis and embryonic tumor incidence of BWS cases may guarantee a more efficient surveillance optimizing tumor screening. As previously reported, a meta-analysis collecting results from seven studies, including 1370 genotyped BWS patients identified 102 cases with BWS-related malignancies (Zhang et al., 1997; Mussa et al., 2016a). It is interesting to note how different was the prevalence among the molecular subtypes: 2.5% (21/836) in KCNQ1OT1:TSS-DMR-LOM, 13.8% (47/341) in UPD, 22.8% (28/123) in H19/IGF2:IG-DMR -GOM and 8.6% (6/70) in patients with *CDKN1C* mutations. Wilms tumor represents the most common cancer in BWS patients (Rump et al., 2005) with a high prevalence in combination with telomeric defects in H19/IGF2:IG-DMR -GOM and UPD subgroups (21.1% vs. 6.2%, respectively, *P* < 0.001); subjects with centromeric defects display a lower rate. The tumor development in H19/IGF2:IG-DMR -GOM cases is significantly higher compared to UPD cases. Adrenal carcinoma has only been observed in UPD (1.5%, *P* < 0.001). Hepatoblastoma development has been associated with UPD (4.7%, *P* < 0.001) (Mussa et al., 2016a) although it is also observed in patients with KCNQ1OT1:TSS-DMR-LOM (0.7%) and H19/IGF2:IG-DMR -GOM (0.8%, *P* < 0.001). Neuroblastic tumors have been correlated with *CDKN1C* mutations (4.3%, *P* = 0.003) despite also observed in KCNQ1OT1:TSS-DMR-LOM (0.5%) and UPD cases (0.9%) with lower prevalence (Brioude et al., 2018). Additional studies are required to implement the available data since some associations seem to be clinically relevant, although not statistically significant (Mussa et al., 2016a). In general, this meta-analysis confirmed previous studies reporting the most common histotypes associated with BWS, such as Wilms tumor, hepatoblastoma, neuroblastic tumors, adrenal carcinoma, and rhabdomyosarcoma (Lapunzina, 2005; Shuman et al., 2006). The overall tumor risk of H19/IGF2:IG-DMR GOM is ∼23%, specifically with a 21% risk of developing Wilms tumor (Maas et al., 2016). The lowest tumor risk regards KCNQ1OT1:TSS-DMR hypomethylated subgroup, although there is a remarkable tumor variability. This subgroup shows different cancer types: hepatoblastoma, rhabdomyosarcoma, and gonadoblastoma but not in Wilms tumor. Patients with H19/IGF2:IG-DMR hypermethylated present Wilms tumor and hepatoblastoma their recurrence is related to *IGF2* overexpression during cancer development (Akmal et al., 1995; Rump et al., 2005; Maas et al., 2016; Mussa et al., 2017; Brioude et al., 2019). Several studies observing different cohorts of BWS patients confirmed higher tumor risk associated with the H19/IGF2:IG-DMR hypermethylated and UPD(11)pat subgroup and high frequency for Wilms tumor and hepatoblastoma (Maas et al., 2016; Ounap, 2016; Brioude et al., 2018; Kamien et al., 2018; MacFarland et al., 2018; Wang et al., 2020). Wilms tumor rate is more frequent in the H19/IGF2:IG-DMR subgroup than in the cases observed for the UPD (Wang et al., 2020). The UPD subgroup is associated with a high prevalence of hemihyperplasia and hepatoblastoma (Maas et al., 2016; Mussa et al., 2017). Only four cases of BWS children belonging to KCNQ1OT1:TSS-DMR hypomethylated subgroup had Wilms tumor or nephrogenic remnants. Adrenocortical tumors have a percentage of 3% in BWS cases, and few of them are associated with the LOM *KCNQ1OT1* gene (Alsultan et al., 2008; Mama et al., 2014). Two cases of *KCNQ1OT1* LOM with adrenocortical tumors also recently observed (Wijnen et al., 2012), and an additional one recently reported, although neither of these patients presented typical phenotypic features of BWS (Wijnen et al., 2012; Eltan et al., 2020). Guidelines are discordant because the European consensus does not recommend this screening (Brioude et al., 2018) that is required in the USA consensus (Kalish et al., 2017).

## Metabolic Imbalance

Metabolic disorders are one of the major clinical conditions in BWS (Schiff et al., 1973). Among metabolic imbalances, hyperinsulinemia/hypoglycemia are pathological states distressing 50% of BWS patients (Martinez y Martinez et al., 1992). Congenital hyperinsulinism (HI) is associated with a dysregulation of insulin secretion from pancreatic β-cells, and the molecular etiology of HI is due to mutations in ABCC8 and KCNJ11 genes located at the 11p15 region encoding for two subunits of the pancreatic β-cell ATP-sensitive potassium channel (KATP channel), SUR1 and Kir6.2 (Tung et al., 2020). Although only a few cases of BWS show HI, 50% of BWS neonates present transient HI while 5% have persistent HI requiring medical and/or surgical management (Senniappan et al., 2015; Kalish et al., 2016). Hypoglycemia can start in the neonatal period during the first days of life (Sweet et al., 2013). Glycemic disorders are related to aberrations of tumor suppressor genes (*IGF2*, *H19*, and *p57KIP2)* located in the 11p15 region and associated with BWS (Lee et al., 1999). Anomalies in the type 1 sulfonylurea receptor (SUR1) gene on chromosome 11p15 have been reported (de Lonlay-Debeney et al., 1999; Glaser et al., 2000). *IGF2* is overexpressed in 20% of BWS individuals (Weksberg et al., 1993), and its loss of imprinting is responsible for hypoglycemia (Lee et al., 1999). Loss of imprinting and UPD on the paternal allele in the 11p15 region (Slavotinek et al., 1997) leads to *IGF2* gene overexpression. IGF2 protein binding to the insulin receptor sustains the hyperinsulinemia condition. It is therefore not surprising that hyperinsulinemic hypoglycemia is often associated with BWS diagnosis. Hypoglycemia BWS cases reported hyperinsulinism and inappropriate insulin secretion (Shepherd et al., 2000). Pancreatic β cell dysregulation has been linked to the cause of hyperinsulinism in BWS (Stanley, 1997). In many histological analyses, hypertrophy and hyperplasia (Lteif and Schwenk, 1999) were observed, strengthening the correlation between incorrect pancreatic activity and BWS (Laje et al., 2013). However, the progression toward type 1 diabetes has not been documented (Leibowitz et al., 1995). The high levels of insulin in the blood cause a prolonged lowering of glucose concentrations, although the mechanism of insulin release in the different secretagogues occurs in different ways (Munns and Batch, 2001). In most BWS patients, life expectancy is good (Weng et al., 1995), and the metabolic imbalance tends to improve within time. However, in some cases, prolonged drug treatment is required to control hypoglycemia (Shilyansky et al., 1997). To obtain better results for glycemic control, it depends upon timeliness (Aynsley-Green et al., 2000). Unfortunately, in 20% of BWS cases, hypoglycemia is difficult to control and may cause severe decompensations leading to neurological alterations with severe repercussions in cognitive function development as intellectual impairment (Cresto et al., 1998). In severe cases, partial pancreatectomy may be required (Martinez y Martinez et al., 1992; Laje et al., 2013).

## Monozygotic Twins Discordance

In twins, it has been observed a discordant monozygotic phenomenon (Weksberg et al., 2002), whereas BWS afflicts one subject, although the other twin may have some characteristics of the syndrome. Theoretically, monozygotic twins (MZ) resulting from a single zygote should have identical genomes. However, several examples of genetic differences have been reported among MZ, thus suggesting that somatic changes may occur after conception (Bliek et al., 2009a). The mosaicism is leading to a discordance between MZ in BWS, and it is linked to an epigenetic event triggered by the twinning process. Cells involved in this event spread among embryos in a multiple pregnancy creating a mosaic distribution being responsible for the variable phenotypic spectrum observed in BWS cases (Cohen et al., 2019). The timing of epigenetic aberrations influences the twinning, the degree of severity of BWS, and the degree of mosaicism. The theory of “diffuse mosaicism” proposed by Cohen et al. (2019) outlines the time points when the epigenetic event occurs in relation to twinning and the determination of chorionicity. In singleton gestations, the epigenetic aberration has been reported to occur first in non-mosaic patients during embryogenesis and subsequently in mosaic patients. In dichorionic gestations, the zygosity determines the timing of the event. It occurs earlier in the dizygotic dichorionic pregnancies than in the monozygotic dichorionic pregnancies (Cohen et al., 2019). Previous research has shown that an epigenetic event before twinning leads to the formation of two different clonal cell populations (Bell and Spector, 2011). These different cell clones repel each other and trigger the twinning event leading to the formation of separate cell masses (Hall, 1996; Machin, 1996; Weksberg et al., 2002). Therefore it is possible that a cell group carrying an imprinting alteration of *KCNQ1OT1* (LOM) could preferably increase its growth rate compared to normal cells, thus generating asymmetry of the entire cell mass and increasing the possibility of cell clones separation genotypically distinct (Weksberg et al., 2002). Most BWS MZ exhibit *KCNQ1OT1* imprinting defects (in KCNQ1OT1:TSS-DMR), indicating that monozygotic twinning is mechanically linked to the imprinting error or, conversely, that epigenetic alterations in KvDMR1 (LOM) can increase the possibility of monozygotic twinning. KCNQ1OT1:TSS-DMR hypomethylation is related to a failure in methylation, and it coincides or occurs shortly after the twinning event (Bliek et al., 2009a; Castillo-Fernandez et al., 2014).

Differences in genotype and phenotype can be attributed to various causes, including non-random inactivation X (Machin, 1996). These pieces of evidence agree with the higher incidence observed in monozygotic discordant female twins, suggesting that monozygotic twinning, genomic imprinting, and X inactivation may be mechanically and temporally related events (Lubinsky and Hall, 1991; Weksberg et al., 2002). In most reports, there is a high prevalence of MZ female and very few cases of discordant male twins (Weksberg et al., 2002; Smith et al., 2006; Bliek et al., 2009a; Tierling et al., 2011). In 250 BWS patients, 20 sets of monozygotic and 2 sets of dizygotic twins with high prevalence (16 out of 20) for female MZ were identified (Weksberg et al., 2002). Later in 2009, another study showed a high MZ twinning rate of 2.5% as compared with 0.3–0.4% among normal twins, while dizygotic (DZ) cases reported 0.75% as rate with 0.7–1.1% of prevalence. A female excess among BWS multiple births was observed in this study. In 10 MZ, 9 were females, and all 3 cases of DZ were females (Bliek et al., 2009a). More recently, in 2019, the high incidence of monozygotic female in a cohort of 26 BWS twins was confirmed (Cohen et al., 2019). The significant female preponderance of the MZ discordant for BWS could be associated with a variety of sex-related factors. For example, the developmental error can occur equally in male and female embryos, demonstrating a lethal effect on male MZ or in alternative the delay of early development in female embryos compared to men (Hall, 1996). It is subordinated to the X inactivation process, and it can increase the susceptibility in female MZ embryos to certain errors. The double discrepancy is due to the failure of Dnmt1o (DNMT1oocyte) to maintain methylation in phase S of a cell cycle occurring before or during the twinning event (Bestor, 2003). It has been hypothesized that the excess of twins in BWS patients is secondary to X inactivation with delayed embryogenesis allowing the acquisition of errors such as failure of maintenance methylation (Lubinsky and Hall, 1991; Orstavik et al., 1995; Hall, 1996; Hall and Lopez-Rangel, 1996; Weksberg et al., 2005). This evidence supports the high incidence of female BWS MZ. In two more extensive studies (Gaston et al., 2001; Weksberg et al., 2002) KCNQ1OT1:TSS-DMR hypomethylation from DNA of blood samples has been reported in affected and unaffected twins of discordant couples. It has been proposed that the aberrant methylation in the blood of the healthy twin is caused by vascular connections in the placenta shared by both MZ (monochorionic, diamniotic). This failure occurs in the eight-cell blastocyst stage preceding the moment when MZ (monochorionic, diamniotic) twinning is established. However, not all methylation defects involve twinning, and most BWS patients are singletons (Cohen et al., 2019). An explanation of these assumptions is related to the theory of endangered twins in which twinning occurs in all cases, but the second fetus is reabsorbed in early pregnancy (Landy and Keith, 1998). The twin discordance of BWS patients is observed not only in females but also in males (Smith et al., 2006). A pair of male MZ discordant for BWS was reported, and among these, the affected twin had paternal UPD for chromosome 11p15. The second male twin pair was concordant, and both demonstrated H19/IGF2:IG-DMR hypermethylation, thus suggesting BWS-related molecular heterogeneity in male MZ (Smith et al., 2006).

## Assisted Reproduction Techniques (ART) and BWS Epigenome

The association between ART and syndromes related to epigenetic defects has been reported in several cases such as BWS (Dhont et al., 1999; Ferraretti et al., 2012, 2013; Kupka et al., 2014), large offspring syndrome in ruminants (LOF) (de Mouzon et al., 2012) and Angelman syndrome (MIM 105830) (Talaulikar and Arulkumaran, 2012) (MRC Working Party on Children Conceived by *In Vitro* Fertilization, 1990). In 1995 a BWS patient conceived through ART (Sutcliffe et al., 1995) was reported, Young et al. (1998) described the LOF, etiologically correlated with *in vitro* fertilization (Kupka et al., 2014). The LOF shows a significant effect on the phenotype connected with ART (Li et al., 2019), and this syndrome is a model for BWS (Chen et al., 2013) presenting similar phenotypic abnormalities (Young et al., 2001). In LOF affected bovines was reported an association between multiple loci imprinted defects and ART. This evidence underlines how ART may cause imprinting disturbances (Mussa et al., 2017). Most of the patients suffering from imprinting disturbances conceived via *in vitro* fertilization (Kupka et al., 2014) and intracytoplasmic sperm injection (ICSI) showed aberrant imprinted DNA methylation (Hattori et al., 2019). In the paper of DeBaun et al. (2003) were identified seven sporadic cases conceived by ART and epigenetic alterations were present in six of them generally associated with BWS. Several studies have further explored this association (Maher and Reik, 2000; Gicquel et al., 2003; Halliday et al., 2004; Chang et al., 2005; Rossignol et al., 2006; Sutcliffe et al., 2006; Doornbos et al., 2007; Lim et al., 2009; Hiura et al., 2012; Tee et al., 2013). The most recent literature also has corroborated the hypothesis that BWS is related to ART (Vermeiden and Bernardus, 2013; Hattori et al., 2019). Indeed, LOM of KCNQ1OT1:TSS-DMR represents the molecular defect found in BWS patients conceived through ART (Gomes et al., 2009; Mussa et al., 2017). In 2015, the clinical study NCT00773825 on the association between BWS and ART was completed, although the results are not yet reported. The authors investigated the methylation status at nine different loci and other epigenetic marks using Southern blot and methyl-specific quantitative PCR in three groups of patients: children naturally conceived, children conceived after ovarian stimulation, but *in vivo* fertilization, and a group of children conceived after ovarian stimulation and *in vitro* fertilization. The aim of this trial was to determine if children born following ART exhibit an increased risk of imprinting defects. Moreover, previous results showed that ART might favor imprinting alterations at the centromeric imprinted 11p15 locus and, consequently, the incidence of BWS. Some of BWS patients reported DNA methylation defects abnormal methylation at loci other than the 11p15 region. This condition was present in both BWS patients naturally, and ART conceived (Gicquel et al., 2008), suggesting that ART procedure could be not specifically involved in the loss of methylation at various imprinting loci (Rossignol et al., 2006).

## Treatment Approaches and Perspectives for Epi-Based Therapies

Currently, there is no dedicated therapy for BWS, and all available treatments are mainly addressing clinical features for ameliorating the quality of life. The current on-going clinical trial NCT01916148 aims to determine the ability F-DOPA PET, a PET radiotracer, to detect focal lesions prior to surgical intervention in BWS patients with hyperinsulinemic hypoglycemia. This study is useful to guarantee an early diagnosis of the pathology and to manage available treatments for BWS patients. Macroglossia occurs in 90% of BWS patients and may regress spontaneously in some children, but 40% of them undergo surgery to reduce tongue size (Brioude et al., 2018). The regional overgrowth in BWS can be progressive or non-progressive (Burkardt et al., 2019). It occurs in 43-60% of patients, and the management is related to the affected limbs (Wang et al., 2020). Leg length discrepancy (LLD) can influence negative life quality and may require shoe lifts or, in some cases, surgical correction (Ghanem et al., 2011; Brioude et al., 2018). The asymmetric overgrowth of the upper limbs generally does not require surgery (Brioude et al., 2018). New strategies are necessary to improve the possible and available treatment for BWS patients (Swinney and Xia, 2014). Many studies have demonstrated that by reprogramming the epigenetic landscape, it is possible to modulate the defects present in the genome leading to the treatment of different diseases (Miranda Furtado et al., 2019). Epigenetic markers can be targeted by activators or inhibitors of epigenetic-modifying proteins (Lauschke et al., 2018), the so-called “epidrugs” currently used mainly in tumor treatments, such as hematological malignancies (Woods et al., 2015; Mazzone et al., 2017). The effectiveness of these treatments is slowly widening toward new fields of application. Epidrugs are demonstrating their potential in other pathologies, such as infectious diseases, metabolic and cardiovascular disorders (Das et al., 2009; Dunn and Rao, 2017). There are promising clinical advances in epigenetics toward new drug discovery (Crea et al., 2011; Glasgow et al., 2015; Lundstrom, 2017) and biomarkers (Shao et al., 2018) in order to limit epigenetic mutation effects (Vitiello et al., 2015; Inamura, 2017). Epigenetic (i.e., hypermethylation of the tumor suppressor gene promoters) and genetic mutations of epigenetic enzymes (loss or gain of function) can be used as predictors of therapy response in different types of diseases (Welch and Clegg, 2010). Prolonged re-expression of epigenetically silenced genes has been demonstrated for various genes, including tumor suppressor genes (Gaur et al., 2015). BWS might represent one of the many new challenges for epigenetic treatment-based applications. For example, it is possible to mitigate CpG island methylation on maternal and/or paternal allele to restore the normal transcriptional activity in the imprinting control regions (Bartolomei and Ferguson-Smith, 2011). Epigenetic treatments might become a valid opportunity for aberrations in 11p15.5 imprinted region (Smith et al., 2012). The Food and Drug Administration (FDA) in the United States has approved several DNA methylation inhibitors, including cytidine analogs 5-azacitidine and zebularine and nucleoside analogs. Histone deacetylase inhibitors (Cheng et al., 2019) such as suberoylanilide hydroxamic acid (SAHA, trade name Vorinostat), romidepsin (trade name Istodax), Valproic acid (VPA) and trichostatin A (TSA) (Kelly et al., 2010; Heerboth et al., 2014) disrupt deacetylation process. New therapeutic programs and technique advancements might be applied to reprogram the epigenetic circuit and to counteract chronic symptoms. Since the main BWS targets are determined by deregulation on epigenetic processes, it should be interesting to evaluate potential targets by using drugs against the activity of DNA methyltransferases and or histone deacetylases and histone acetylation. These drugs should be potential treatments against BWS. Ideally, the first application may be a combination therapy to control disease progression. For example, surgical techniques for phenotypic abnormalities control (Wang et al., 2020) might be supported by the use of epi-based treatments for the metabolic imbalances in young BWS patients. The epigenetic sensitization to radiotherapy might provide promising results in BWS affected by Wilms tumor. Moreover, given the potential therapeutic role of epigenetic modulating agents in metabolic disorders (Crispo et al., 2019), it is tempting to hypothesize that in the near future, by targeting epi-modifiers and remodelers might prove beneficial also in BWS. Clearly, given the genome and epigenome heterogeneity and complexity of this disease, patient’s stratification may represent a ‘*conditio sine qua non*’ for future epi-based applications.

## Conclusion

The progress in epigenetic drugs discovery (Morera et al., 2016; Esteller, 2017; Velasco and Francastel, 2019), the involvement of epigenetic mutations in a wide range of diseases (Dirks et al., 2016; Graca et al., 2016; Jones et al., 2016; Berdasco and Esteller, 2019) and the newly chromatin-based identified disease biomarkers (Dirks et al., 2016) have suggested epi-based approaches as a promising tool for clinical applications. Evidently, potential new therapeutic options require better clinical knowledge. Indeed, the target identification and characterization are at the basis for a correct therapy. In the case of BWS there are some levels of complexity to be decrypted. Being a rare disease, the generally low number of patients is a bottleneck and a hindrance to the development of dedicated therapeutic approaches nor the causal identification of “druggable” targets proven beneficial for the restoration of the health status or, at least, for symptoms defeat. The lesson learned from BWS homozygotic twins not only suggests the existence of a phenotypic link between epigenome deregulation and the complexity of BWS disease, but it also indicates the potential of targeting the epigenome pharmacologically in BWS to at least obtain beneficial effects ameliorating the quality of life. Indeed, on one side, the different disease phenotypes in homozygotic BWS twins have consolidated the idea that the disease penetrance is epigenome-regulated, despite somatic changes may occur after conception. On the other side, this proves in humans the potential of a therapeutic approach targeting BWS epigenome. Recently we have just started to understand the pivotal role of epigenome deregulation in BWS (and other rare diseases) supporting the development of diagnostic, prognostic, and therapeutic approaches also based on this notion. In this perspective, therapeutic approaches might also be applied to epimutations related to tumor predisposition in BWS, which might gain benefit from the use of treatment schemes, including or based on chromatin acting drugs.

## Author Contributions

CP, UC, RB, and LA: conceptualization. LA and RB: funding acquisition. CP, UC, MN, and RB: writing – original draft preparation. RB, LA, and MN: writing – review and editing. MN: writing and editing. All authors contributed to the article and approved the submitted version.

## Conflict of Interest

The authors declare that the research was conducted in the absence of any commercial or financial relationships that could be construed as a potential conflict of interest.

## Abbreviations

ART: assisted reproductive technologies
BWS: Beckwith-Wiedemann syndrome
BWSp: Beckwith-Wiedemann syndrome spectrum
CNVs: copy number variations
CTCF: CCCTC-binding factor
DMR: differentially methylated region
DZ: dizygotic twins
GOM: gain of methylation
IG-DMR: intergenic differentially methylated region
ICR1: imprinting control region 1
ICR2: imprinting control region 2
LLD: leg length discrepancy
LOF: large offspring syndrome
LOM: loss of methylation
MS-MLPA: methylation-specific multiplex ligation-dependent probe amplification
MZ: monozygotic twins
ncRNA: non-coding RNA
TSS-DMR: transcription start site differentially methylated region
UPD: uniparental disomy.
